# In Plasma Catalytic Oxidation of Toluene Using Monolith CuO Foam as a Catalyst in a Wedged High Voltage Electrode Dielectric Barrier Discharge Reactor: Influence of Reaction Parameters and Byproduct Control

**DOI:** 10.3390/ijerph16050711

**Published:** 2019-02-27

**Authors:** Juexiu Li, Hongbo Zhang, Diwen Ying, Yalin Wang, Tonghua Sun, Jinping Jia

**Affiliations:** 1 School of Environmental Science and Engineering, Shanghai Jiao Tong University, Shanghai 200240, China; lijuexiu@sjtu.edu.cn (J.L.); hongbo_zhang888@163.com (H.Z.); yingdw@sjtu.edu.cn (D.Y.); ylwf@sjtu.edu.cn (Y.W.); sunth@sjtu.edu.cn (T.S.); 2 Shanghai Institute of Pollution Control and Ecological Security, Shanghai 200092, China

**Keywords:** VOCs, in plasma catalytic, dielectric barrier discharge, CuO foam, toluene

## Abstract

Volatile organic compounds (VOCs) emission from anthropogenic sources has becoming increasingly serious in recent decades owing to the substantial contribution to haze formation and adverse health impact. To tackle this issue, various physical and chemical techniques are applied to eliminate VOC emissions so as to reduce atmospheric pollution. Among these methods, non-thermal plasma (NTP) is receiving increasing attention for the higher removal efficiency, non-selectivity, and moderate operation, whereas the unwanted producing of NO_2_ and O_3_ remains important drawback. In this study, a dielectric barrier discharge (DBD) reactor with wedged high voltage electrode coupled CuO foam in an in plasma catalytic (IPC) system was developed to remove toluene as the target VOC. The monolith CuO foam exhibits advantages of easy installation and controllable of IPC length. The influencing factors of IPC reaction were studied. Results showed stronger and more stable plasma discharge in the presence of CuO foam in DBD reactor. Enhanced performance was observed in IPC reaction for both of toluene conversion rate and CO_2_ selectivity compared to the sole NTP process at the same input energy. The longer the contributed IPC length, the higher the toluene removal efficiency. The toluene degradation mechanism under IPC condition was speculated. The producing of NO_2_ and O_3_ under IPC process were effectively removed using Na_2_SO_3_ bubble absorption.

## 1. Introduction

Volatile organic compounds (VOCs) emission and elimination have become hot issues in recent years. Large quantity of VOC emissions were produced by fugitive anthropogenic emission from industrial processes and chemical products [1]. Industrial processes like thermal power, coking, and iron smelt were often accompanied with NO_x_ and SO_2_ emissions [2,3]. In addition to the adverse health effect on the respiratory system and carcinogenesis risks [4,5,6,7,8], VOC emission contributed to regional air pollution [9,10]. VOCs were also major precursors of ozone (O_3_) and secondary organic aerosols (SOA) [11,12,13]. Taking toluene as an example, smog chamber studies showed SOA production and yields were much higher under urban ambient air against that with purified air when toluene coexisted with NO_x_ and SO_2_ [14]. The presence of toluene also promotes aerosol nucleation and growth process with coexistence of SO_2_ [15]. Therefore, it is urgent to reduce VOC emissions from flue gas before discharge into atmosphere.

The massive fossil fuel and energy consumption leads to the innovation of VOC control technologies, especially in China. The development of renewable resources for energy supply is both a sustainable and long-term solution for VOC emission control from the root. Currently, many physical and chemical methods have been developed to reduce VOC emissions, and these methods mainly include physical adsorption [16], biodegradation [17,18,19], thermal catalytic oxidation [20,21], hybrid membrane/condensation [22], and combined technology [23,24]. Among these emerging strategies, the adsorption technology exhibits the advantages of low cost, easy operation and no increase of CO_2_ emissions. However, the major obstacle of adsorption is the tough recyclability of the absorbents. Biodegradation exhibited low cost and easy operation, but the VOC removal efficiency and stability remains at lower level. The thermal oxidation featured high VOC removal efficiency and good stability, whereas high operation and maintenance cost limited the applications. The catalytic oxidation of VOCs was also attractive, such as a ceramic monolith platinum-rhodium three-way converter used on gasoline vehicle exhaust treatment. The main obstacles of catalytic oxidation are the high cost of noble metals and strict application environment. Recently, VOC decomposition with the assistance of non-thermal plasma (NTP) has become increasingly attractive because of the moderate operation conditions (ambient temperature and atmospheric pressure), rapid start-up, and non-selective oxidation of target molecules [25,26]. NTP also exhibits good performance on removal of SO_2_, NO_x_, and Hg^0^ [27,28,29]. Nevertheless, the energy efficiency and CO_2_ selectivity towards VOC abatement remain at a lower state. To tackle this issue, heterogeneous catalyst was often introduced into the discharge zone to form an in plasma catalysis (IPC) reaction in a dielectric barrier discharge (DBD) reactor.

Various catalysts, including metal oxides [30] and perovskite [31], are often loaded on γ-Al_2_O_3_ or zeolite to perform the plasma-catalytic process [32]. However, the powder- or pellet-loaded catalyst remains inconvenient to fill into the reactor. Additionally, a large pressure drop will be produced on condition of a longer plasma region and larger gas flow rate after catalyst loading. From this aspect, we consider filling the plasma region with a foam structure catalyst, which exhibits the advantages of reducing gas flow resistance and obtaining desirable gas permeability. The foam structure can also provide a very large number of surface active sites for gas diffusion and mass transfer. Moreover, the monolith foam structure catalyst can be easily installed and reused. Few studies investigated foam structural catalysts in plasma reaction. Guo et al. developed four metal oxides deposited on alumina/nickel foam (NF) and found that in situ combination of plasma with catalyst can improve toluene removal efficiency, CO_2_ selectivity, and deduction of unwanted byproducts [33,34].

Based on the above discussion, this paper investigated abatement of VOCs in a wedged high electrode tubular DBD reactor using monolith CuO foam as catalyst. Toluene was used as the target VOC molecule owing to its wide application and vast emission [35]. The CuO foam was prepared via an annealing process of copper foam. Monolith CuO foam exhibits the convenience of installation and controllability of the IPC length so as to more effectively decompose VOC molecules. The CuO foam surface morphology and crystalline structure before and after usage were characterized. The influencing factors of IPC reaction (including catalyst loading, input peak voltage, gas flux, toluene initial concentration, and relative humidity) were investigated and optimized. The toluene degradation mechanism was speculated based on gaseous intermediates evaluation by gas chromatography with time-of-flight mass (GC-TOF-MS) measurement. The control of toluene degradation byproducts under the IPC process, i.e., NO_2_ and O_3_, were also preliminarily studied using Na_2_SO_3_ bubble absorption.

## 2. Materials and Methods

### 2.1. Experimental Setup of the IPC System

**Figure 1:**
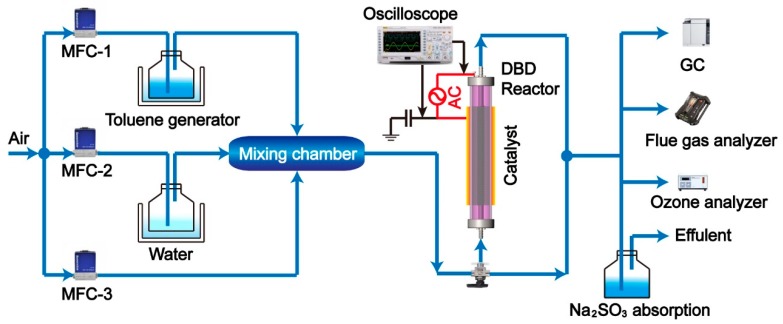
Schematic diagram of in plasma catalytic configurations for toluene decomposition.

The IPC reaction system consisted of toluene generation, dielectric barrier discharge region, and tail gas detection unit (as shown in Figure 1). Gaseous toluene was produced via buffer strategy with pure air as carrier gas. Specifically, air passed through a bottle containing pure toluene chilled in an ice−water isothermal bath to obtain a given toluene concentration. Water vapor was purged to investigate its influence on toluene removal. VOCs streams were well premixed in a mixing chamber prior to the discharge region, thus giving a fixed inlet toluene concentration of 1000 ppm and flux of 400 mL/min without specific illustration.

The DBD reactor was coaxial type with tubular quartz tube (>99.9% SiO_2_, dielectric constant: 3.75) as the discharge barrier, and with total length of 300 mm and inner diameter of 20 mm. Stainless steel mesh with a variable length of 5, 10, and 15 cm wrapped outside the quartz tube acted as ground electrode to achieve the discharge volume varying from 8.0, 16.0, and 24.0 cm^3^. A wedged stainless steel rod with a diameter of 14 mm was end-fixed along the axis of the cylinder and acted as a high-voltage electrode. The wedged configuration of the corona electrodes was 1 mm in height. More detailed illustration of the wedged high electrode was shown in Figure S1. As with the decrease of discharge gap showed a better VOCs removal performance [36], the discharge gap in this study was fixed at 3 mm.

The plasma discharge was driven by a high voltage alternating current (AC) power (sine wave, 5–20 kHz, 0–0 kV, Suman Co., Nanjing, China). The electro-parameters of the reactor were monitored by an oscilloscope (DS5062MA, Rigol, Suzhou, China). The consumed energy of the adjacent dielectric barrier was calculated from Lissajous figures, using a 1 μF capacitor inserted between the reactor and the ground. The discharge power added on toluene removal were valued by applied peak voltage and specific input energy (SIE, J/L), with peak voltage varied from 8 to 24 kV corresponding to SIE varying from 70.8 to 856 J/L (as shown in Table S1). The frequency of the AC power was set at a fixed frequency of 7.5 kHz according to the intrinsic character of the high voltage power supply (shown in Figure S2).

### 2.2. Preparation and Characterization of CuO Foam

CuO foam used in this study was prepared using a simple annealing process. Briefly, 3 mm thickness of copper foam (purity > 99.8%, Kunshan Jiayisheng Electronics Co., Kunshan, China) was manually rolled to a hollow cylindrical shape with different length and fixed external diameter of 20 mm. The tailored copper foam was then pretreated successively with 0.1 M HCl aqueous solution and ethyl alcohol upon ultrasonic vibration for 20 min to remove impurities. The thus-obtained copper foam was washed with deionized (DI) water and ethanol several times before it was dried at 105 °C for 12 h. Finally, the CuO foam was fabricated in situ over the copper foam through a heating process in a tubular furnace at 550 °C under an air atmosphere for 6 h with a heating rate of 5 °C/min from 25 °C. The thus-prepared monolith CuO foam catalyst can be easily filled and removed from the DBD reactor.

The grain morphology of the prepared CuO foam was investigated by field emission scanning electron microscopy (FESEM, Sirion 200, ThermoFisher, Hillsboro, OR, USA) with a resolution of 3.0 nm at 5 kV, 2.0 nm at 10 kV, and 1.5 nm at 15 kV. The phase structural characterization of CuO foam was further analyzed by X-ray diffractometer (XRD-6100, Shimadzu, Kyoto, Japan) using Cu Kα radiation, respectively. The XRD patterns were acquired in the diffraction angle range from 20 to 90° with a scanning rate of 2 ^o^/min.

### 2.3. Toluene Removal and Evaluation

Before the plasma catalytic reaction, the toluene stream was fed through the DBD reactor for 10 min to achieve saturated absorption (as shown in Figure S3). The gaseous products after toluene IPC reaction mainly included toluene, carbon oxides (CO and CO_2_) and byproducts (NO_x_, O_3_, and organic intermediates). The inlet and outlet concentration of toluene were analyzed by a gas chromatograph (GC-2010, Shimadzu, Kyoto, Japan) equipped with a flame ionization detector (FID) and with an Rtx-1 column. The gas chromatograph column oven temperature was held at 100 °C, with injector and detector temperatures of 200 °C. CO_2_ was measured using a gas chromatograph flame ionization detector with a methanizer (GC-950, Haixin Co., Shanghai, China) and with TDX-01 packed molecular sieve column. The methanizer temperature was held at 350 °C, the column oven temperature was 80 °C, and the injector and detector temperature was 200 °C. The quantification of gas phase toluene and CO_2_ were carried out by using external standard method. The organic intermediates were further analyzed using gas chromatography with time-of-flight mass (GC-TOF-MS, with Agilent GC 7890A and LECO PEGASUS high throughput TOF-MS, Saint Joseph, MI, USA). The GC column was DM-FAMEWAX with diameter of 0.25 µm and length of 30 m. NO_x_ and O_3_ generated by the DBD reaction were monitored by a flue gas analyzer (Testo 340, Testo, Lenzkirch, Germany) and an ozone monitor (UV 300B, Limei Co., Guangzhou, China), respectively. All experiments were conducted at least three times (*n* ≥ 3) and data reported were mean values.

Toluene conversion rate (*R*_T_, %), CO_2_ selectivity (*S*CO_2_, %), and the specific input energy (SIE) were used to evaluate different processes and calculated as follows:

(1) $$R_{T}=\frac{{\left[\mathrm{toluene}\right]}_{\mathrm{in}}-{\left[\mathrm{toluene}\right]}_{\mathrm{out}}}{{\left[\mathrm{toluene}\right]}_{\mathrm{in}}}\times 100,\;\%$$

(2) $$S{\mathrm{CO}}_{2}=\frac{{\left[{\mathrm{CO}}_{2}\right]}_{\mathrm{out}}}{7\times \left({\left[\mathrm{toluene}\right]}_{\mathrm{in}}-{\left[\mathrm{toluene}\right]}_{\mathrm{out}}\right)}\times 100,\;\%$$

(3) $$\mathrm{SIE}\;(J/L)=\frac{\mathrm{Discharge}\;\mathrm{power}}{\mathrm{Gas}\;\mathrm{flow}\;\mathrm{rate}}\times 60$$

where [toluene]_in_ and [toluene]_out_ are the toluene inlet and outlet concentration, and [CO_2_]_out_ is the CO_2_ outlet concentration, respectively. Discharge power unit: W, gas flow rate unit: L/min.

## 3. Results and Discussion

### 3.1. Characterization of CuO Foam

#### 3.1.1. XRD of CuO Foam

**Figure 2:**
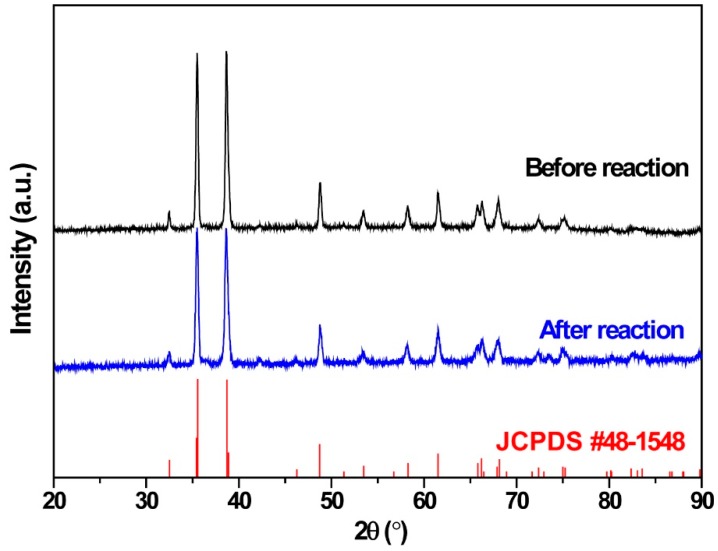
XRD patterns of CuO foam before and after IPC reaction with Joint Committee on Powder Diffraction Standards (JCPDS) data.

As shown in Figure 2, the prepared CuO foam exhibited sharp diffraction peaks which were well matched with Joint Committee on Powder Diffraction Standards (JCPDS) card no. 48-1548 [37]. The diffraction patterns indicated that CuO nanoparticle was single crystalline and there were no other peaks related to Cu_2_O, confirming the complete oxidation of Cu foam during preparation and good phase purity. Additionally, the CuO crystalline structure exhibited no phase change and slight decrease of diffraction peaks after 50 cycles of IPC reaction, indicating the stability of CuO foam under plasma reaction and slight surface corrosion of CuO particle caused by toluene decomposition byproducts.

#### 3.1.2. FESEM of CuO Foam

**Figure 3:**
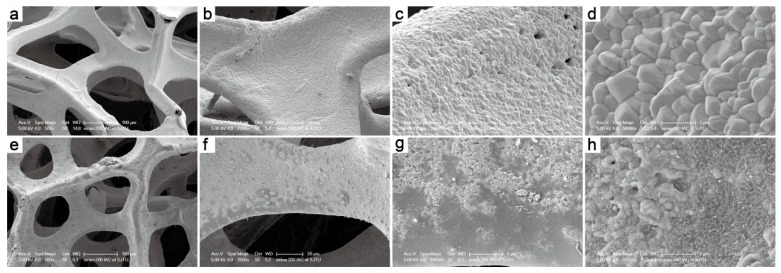
FESEM images of CuO foam before (**a–d**) and after (**e**–**h**) IPC reaction. (With magnification of 500 (**a,e**), 2000 (**b,f**), 10000 (**c,g**) and 50000 (**d,h**)).

Figure 3 shows the morphology of the CuO foam before and after IPC toluene reaction characterized by FESEM. It is observed that the foam framework shown no obvious change after annealing preparation previously described. CuO nanoparticles (with average size of 200–500 nm, Figure 3d) uniformly grew on the framework without any holes. Notably, the CuO grain surface was partly covered by oil-like substance after the IPC reaction (Figure 3h), which was the surface corrosion caused by toluene degradation intermediates. This phenomenon was also proved by the XRD results, in accordance with the slight decrease of CuO diffraction peaks. The granularity of the CuO grain after the IPC reaction becomes smaller and slightly obscure, leading to more uniform distribution and higher dispersion. Meanwhile, it was apparent that the oil-like substance on the inner tube wall was much less than that of the NTP reaction (as shown in Figure S4), confirming that the toluene degradation byproduct was mainly adsorbed by the CuO framework in the IPC reaction [38].

### 3.2. Effect of Catalyst Loadings and Applied Power

**Figure 4:**
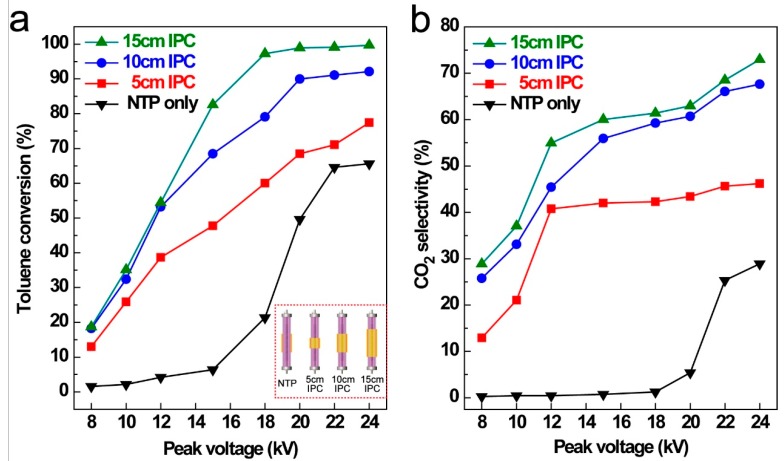
Toluene conversion (**a**) and CO_2_ selectivity (**b**) with various catalyst loadings and absence of catalyst as a function of applied peak voltage. (reaction condition: toluene initial concentration = 1000 ppm, flux = 400 mL/min, RH = 0%, ambient temperature = 25 °C).

The oxidation capacity of IPC system comparing with that of NTP only process for toluene decomposition was investigated at steady-state conditions and results were listed in Figure 4. It was obvious that the plasma discharge became more intense and uniform under IPC condition comparing with that of NTP process (shown in Figure S5). It was also noticed that breakdown voltage was 18 kV for the NTP process, and was only 12 kV for the IPC process. Only 65.6% of toluene conversion (*R*_T_) and 28.9% of CO_2_ selectivity (*S*CO_2_) were obtained at maximum peak voltage of 24 kV under the sole NTP condition. Enhanced *R*_T_ and *S*CO_2_ at the same input peak voltage were obtained after CuO foam catalyst filling into plasma region (IPC) compared with that of sole NTP process. With the IPC region length increasing from 5 cm to 15 cm, both *R*_T_ and *S*CO_2_ increased simultaneously. The longer of the IPC length leaded to a larger discharge volume thus enhancing the toluene oxidation capacity. The maximum *R*_T_ and *S*CO_2_ were achieved at 15 cm of IPC length and 24 kV of peak voltage with *R*_T_ = 99.7% and *S*CO_2_ = 72.9%, respectively. Notably, *S*CO_2_ showed no obvious increase at 5 cm of IPC length when peak voltage was higher than 15 kV (40.8% to 46.2% at 24 kV), whereas the *R*_T_ maintained an increasing trend.

It is well-known that high-energy electrons produced by plasma discharge can cleave the bond between between the methyl group and the aromatic ring (5.0–5.3 eV) [39,40], whereas the dissociation energy of C=C in the aromatic ring is beyond 5.4 eV [41]. At 20 kV of peak voltage, the toluene conversion reached 98.9 % at IPC length of 15 cm, and 90.0% at 10 cm of IPC length, 68.5% at 5 cm of IPC length compared with that of 49.6% under the sole NTP process. The mechanism of toluene degradation under IPC reaction will be discussed after further analysis.

### 3.3. NO_x_ and O_3_ Production

The main drawbacks of non-thermal plasma were the unavoidable production of ozone (O_3_) and nitric oxides (NO_x_) once discharge using air as carrier gas (the generation processes were illustrated as Equation (4)–(8)). Briefly, the atomic oxygen produced by O_2_ collision recombined with O_2_ molecule, which leaded to the O_3_ formation. The massive active nitrogen free radicals produced by N_2_ collision recombined with O_2_, thus causing the production of NO_x_.

**Figure 5:**
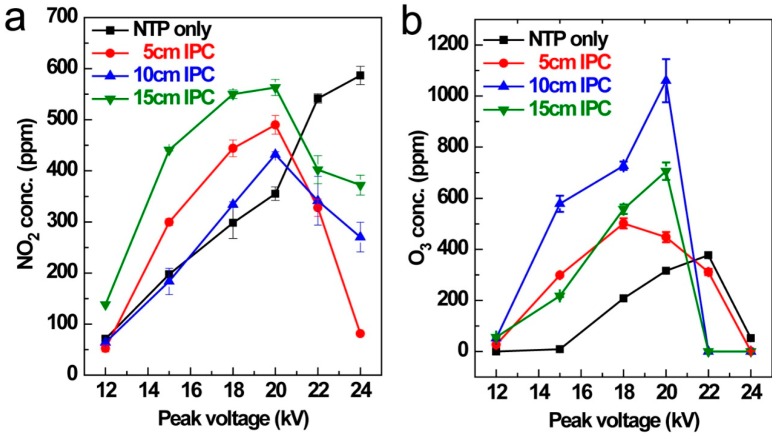
NO_2_ (**a**) and O_3_ (**b**) production under different catalyst loading and input peak voltage. (IPC length = 15 cm, RH = 0%, toluene initial concentration = 1000 ppm, flux = 400 mL/min, ambient temperature = 25 °C).

The influence of the input energy on O_3_ and NO_x_ formation was presented in Figure 5. As NO is readily oxidized to NO_2_ by abundant ozone after discharge, the major NO_x_ species detected in this study was NO_2_ for both IPC and sole NTP process. In general, higher input peak voltage led to increasing production of NO_2_ and O_3_ when peak voltage increased from 12 kV to 20 kV. After the applied peak voltage was higher than 20 kV, NO_2_ concentration in IPC process started to decrease. The reason can be attibuted to NO_2_ reduction by active nitrogen free radical under stronger plasma discharge condition (Equations (9) and (10)). The NO_2_ concentration for the NTP process maintained an increasing tendency with the input voltage ranging from 12 to 24 kV, which was attributed to the lower energy density comparing with that of IPC process. The O_3_ concentration also showed deceasing trend after the maximum concentration when the input peak voltage was higher at all conditions. The ozone concentration of both NTP and IPC reactions decreased to nearly zero when the peak voltage reached 24 kV, which means that the ozone dissociation reactions become dominant at higher energy input. With the increase of energy density, the plasma-forming gas temperature was slightly increased, which also contributed to O_3_ decomposition to a certain extent [42,43].

(4) $$O_{2}+e\;\to \cdot O+\cdot O$$

(5) $$O_{2}+\cdot O+M\;\to \;O_{3}$$

(6) $$N_{2}+e\;\to \;\cdot N+\cdot N$$

(7) $$O_{2}+\cdot N\;\to \;\mathrm{NO}+\cdot O\;\to \;{\mathrm{NO}}_{2}$$

(8) $$O_{3}+\mathrm{NO}\;\to \;{\mathrm{NO}}_{2}+O_{2}$$

(9) $${\mathrm{NO}}_{2}+\cdot N\;\to \;2\mathrm{NO}$$

(10) $$\mathrm{NO}+\cdot N\;\to \;N_{2}+\cdot O$$

### 3.4. Influence of VOC Initial Concentration and Stream Flux

**Figure 6:**
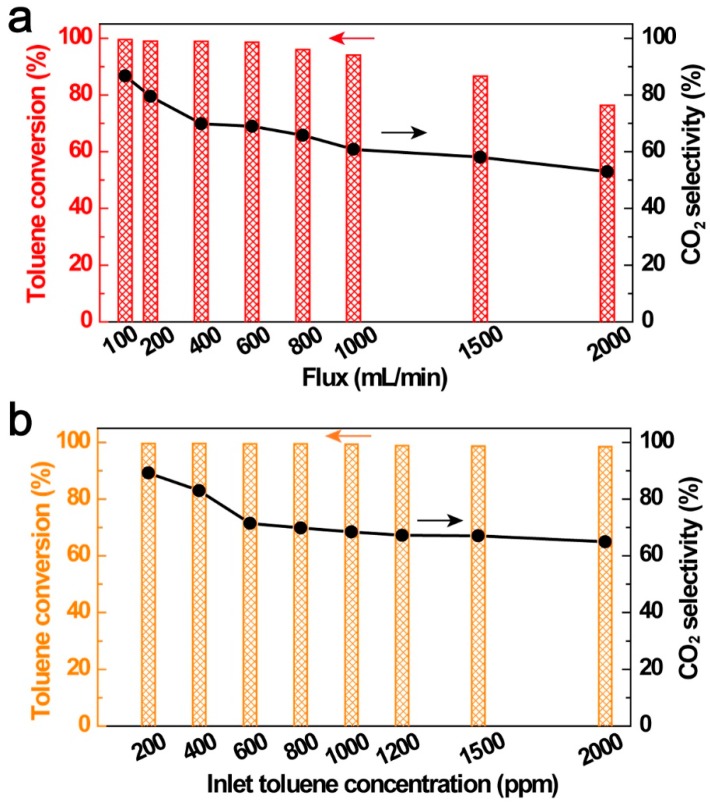
Influences of flux (**a**) and inlet toluene concentration (**b**) on toluene conversions. (bar: toluene conversion, dot line: CO_2_ selectivity) (IPC length = 15 cm, RH = 0%, ambient temperature = 25 °C).

The VOC stream flux and concentration usually fluctuated during emission. Therefore, the influences of VOC stream flux and concentration should be taken into consideration because it directly reflected the capability of plasma screening VOCs efficiency. As shown in Figure 6a, the toluene initial concentration was fixed at 1000 ppm, with the increase of toluene flux from 100 mL/min to 2000 mL/min, both toluene conversion efficiency and CO_2_ selectivity showed a decreasing tendency. At the maximum flux of 2000 mL/min, *R*_T_ and *S*CO_2_ decreased to 76.4% and 52.9%, respectively, comparing with that of 99.8% and 86.9% at a minimum flux of 100 mL/min. With the increase of the VOC stream flux, the gas retention time decreased, then leading to a decreasing number of active radicals reaction with toluene molecules and reducing collision probability with electrons. The influence of toluene initial concentration was shown in Figure 6b with a fixed flux of 400 mL/min, the *R*_T_ kept relative steady and achieved 98% at maximum toluene initial concentration of 2000 ppm, while the *S*CO_2_ decreased significantly when toluene concentration increased from 200 ppm to 600 ppm. *S*CO_2_ kept relatively steady when toluene concentration was higher than 600 ppm, and reached 66% at 2000 ppm. The influences of VOC stream flux and inlet concentration indicated the high removal capacity of the CuO foam IPC system for both endurance of variable toluene concentrations and flux.

### 3.5. Influence of Relative Humidity (RH)

**Figure 7:**
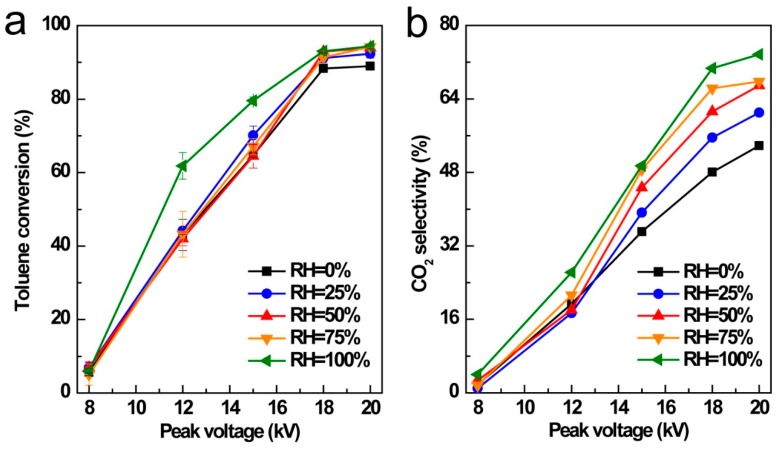
Influence of relative humidity on toluene conversion (**a**) and CO_2_ selectivity (**b**).

Flue gas from industry process usually contains variable water content. Herein, the influence of water vapor was investigated by bubbling air through water thus giving variable relative humidity (RH). As shown in Figure 7, enhanced toluene conversion and CO_2_ selectivity were achieved at higher relative humidity. Notably, obvious toluene conversion enhancement was obtained from 12 to 15 kV under saturated water vapor (RH = 100%), comparing with that of unsaturated water vapor conditions (RH = 0%, 25%, 50%, and 75%). Some relevant studies also showed the same phenomenon as for the humidity influence [44,45].

It is well known that direct electron impact and radical attack are the dominant degradation mechanisms for toluene molecules decomposition by NTP technology. Under higher humidity, increasing hydroxyl radicals were produced in the presence of water vapor because of the electron reaction with H_2_O molecule (Equation (11)), thus leading to the enhancing oxidation ability and higher decomposition rate of toluene molecules. In plasma discharge process, oxygen molecules react with high-energy electron to generate oxygen radicals. The free oxygen radical (·O) also attack H_2_O thus leading to the generation of •OH radicles (Equation (12)):

(11) $$e+H_{2}O\;\to \;\cdot \mathrm{OH}+\cdot H$$

(12) $$\cdot O+H_{2}O\;\to \;2\cdot \mathrm{OH}$$

### 3.6. Degradation Mechanism

**Figure 8:**
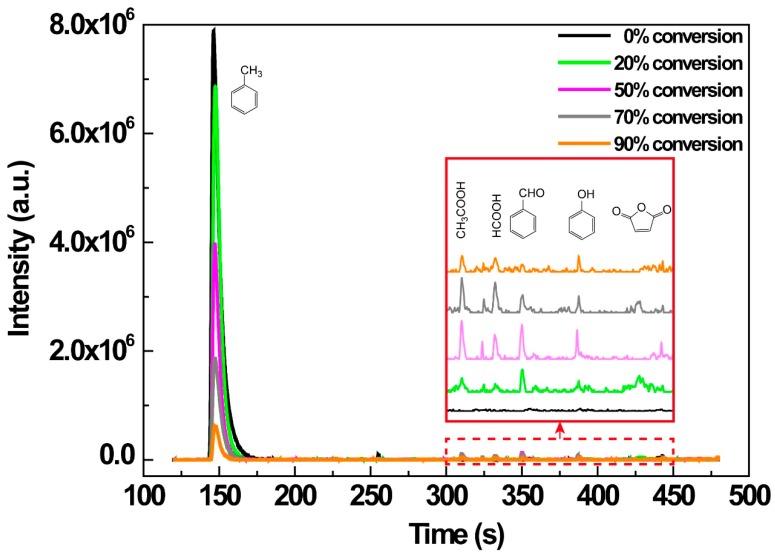
TIC diagrams of main toluene degradation intermediates under different toluene conversions using GC-TOF-MS.

The reaction mechanisms of VOCs decomposition by NTP are complicated because NTP is unique in that it induced various non-equilibrium chemical reactions besides direct dissociation of VOC molecules by energetic electrons [25]. For further understanding of the mechanism of the toluene degradation in IPC reaction, the gaseous toluene degradation intermediates were analyzed using GC-TOF-MS at different toluene conversions. Figure 8 showed the total ion chromatogram (TIC) of the gaseous organic intermediates with the toluene conversion of 20%, 50%, 70%, and 90%, respectively. It is clear to see that, except for toluene, the main following organic by-products were detected: formic acid, acetic acid, benzaldehyde, maleic anhydride, and phenol, while the concentrations of those by-products were much lower than that of toluene. With the promotion of toluene conversion, increases of benzaldehyde, maleic anhydride, acetic acid, and formic acid were observed. When the *R*_T_ was higher than 90%, the organic byproducts decreased significantly, and CO_2_ accounted for the majority.

It is generally accepted that the energetic electrons and a large quantity of radicals (•O, •N, and •OH, et al.) produced by discharge dominated the toluene destruction, whereas the contributions of O_3_ directly reacted with toluene and ion collisions were negligible [33]. During the toluene oxidation reaction, the activated sites belonged to the C–H bond on the methyl group of toluene. The C–H bond dissociation energy of the phenyl group and methyl group are 110 and 75 kcal/mol, respectively, and benzene is relatively difficult to be oxidized compared with toluene [46,47]. Combining with the above intermediates analysis, we speculated the toluene degradation mechanism under IPC reaction: toluene was decomposed and underwent a series of consequent reactions: toluene → benzaldehyde → benzoic acid → chain carboxylic acids (maleic anhydride) → formic acid and acetic acid → CO_2_.

### 3.7. Byproducts Control

**Figure 9:**
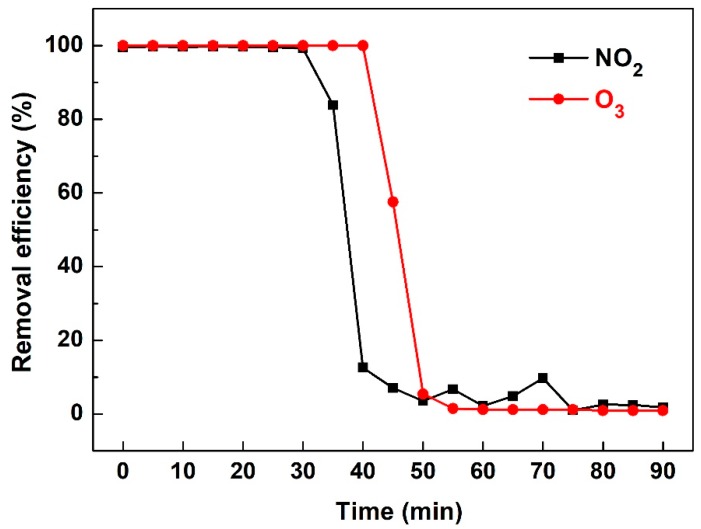
Elimination of NO_2_ and O_3_ using Na_2_SO_3_ absorption (0.5%, wt%).

As shown in Figure 5, the unavoidable production of O_3_ and NO_x_ after the discharge process is a major drawback of the non-thermal plasma reaction under air atmosphere. Herein, Na_2_SO_3_ bubble absorption was preliminarily utilized to remove NO_2_ and O_3_ produced in the plasma reaction (Figure 9). The initial NO_2_ and O_3_ levels after the plasma discharge process were fixed at 480 ppm and 330 ppm, respectively. It can be concluded that NO_2_ and O_3_ were completely absorbed by Na_2_SO_3_ in the initial 40 min, and the promotion of Na_2_SO_3_ absorption concentration and the increase of pH value will prolong NO_2_ and O_3_ removal effectiveness [48]. The post sodium-based alkali solution absorption is an effective application of the NTP technique in industrial flue gas treatment.

## 4. Conclusions

In this study, CuO foam was prepared and was taken as IPC reaction catalyst for the oxidation of gaseous toluene. The influencing factors of IPC reaction were studied. The monolith CuO foam catalyst featured easy installization, controllable plasma catalytic region length, and good gas permeability compared with powder catalysts. Results showed that, in the presence of CuO foam, obvious promotion of plasma energy and a synergistic effect were obtained under IPC reaction, then led to enhanced toluene conversion and CO_2_ selectivity compared with the sole NTP process. Both of the CuO foam framework and phase structure maintained good stability after cycles of IPC reaction. The maximum toluene conversion and CO_2_ selectivity were achieved at 15 cm of IPC length and peak voltage of 24 kV with *R*_T_ = 99.7% and *S*CO_2_ = 72.9%, respectively. The increase of the IPC region and relative humidity led to enhanced toluene conversion. The possible reaction pathway for toluene decomposition was also speculated based on the analysis of gaseous organic byproducts. In view of future application of NTP techniques in flue gas treatment, the post sodium-based alkali solution absorption can be combined with NTP for its effective control of unwanted NO_2_ and O_3_.

## Supplementary Materials

The following are available online at https://www.mdpi.com/1660-4601/16/5/711/s1, Table S1: Specific input energy (SIE) with different input power and peak voltage. Figure S1: Enlarged illustration of the wedged high electrode and DBD reactor. Figure S2: Waveforms of applied voltage and V-Q Lissajous diagrams of IPC process at 15 kV peak voltage. Figure S3: Toluene adsorption balance of different CuO foam loading in IPC reactor. Figure S4: Toluene decomposition byproduct on inner barrier tube comparison of NTP and IPC process. Figure S5: Discharge phenomenon of DBD process with (right view) and in the absence of CuO foam (left view) as catalyst.
