# Supplementary material for: In Plasma Catalytic Oxidation of Toluene Using Monolith CuO Foam as a Catalyst in a Wedged High Voltage Electrode Dielectric Barrier Discharge Reactor: Influence of Reaction Parameters and Byproduct Control
Source: Int J Environ Res Public Health. 2019 Feb 27;16(5):711. doi: 10.3390/ijerph16050711 (PMC6427108; doi:10.3390/ijerph16050711)
Supplement: Supplementary file 1 [file ijerph-16-00711-s001.pdf]

Supplementary file

In Plasma Catalytic Oxidation of Toluene Using Monolith CuO Foam as a Catalyst in a Wedged High Voltage Electrode Dielectric Barrier Discharge Reactor: Influence of Reaction Parameters and Byproduct Control

Juexiu Li[1], Hongbo Zhang[1], Diwen Ying[1], Yalin Wang[1], Tonghua Sun[1], Jinping Jia[1,2,*]

1 School of Environmental Science and Engineering, Shanghai Jiao Tong University, No. 800 Dongchuan Road, Shanghai 200240, China; lijuexiu@sjtu.edu.cn (J. L.), hongbo_zhang888@163.com (H. Z.); yingdw@sjtu.edu.cn (D. Y.); ylwf@sjtu.edu.cn (Y. W.); sunth@sjtu.edu.cn (T. S.)

2 Shanghai Institute of Pollution Control and Ecological Security, Shanghai 200092, China

* Correspondence: jpjia@sjtu.edu.cn (J. J.); Tel: +86-21-54742817

* Corresponding author.

Email address: jpjia@sjtu.edu.cn

Tel: +86-21-54742817; Fax: +86-21-54742817;

Table S1. Specific input energy (SIE) with different input power and peak voltage.

**Peak voltage (kV)**	8	10	12	15	18	20	22	24
**SIE (J/L)**	70.8	115.6	151.2	332.2	602.6	696.5	799.8	856
**Input power (W)**	6.9	8.8	12.5	20.2	37.5	45.2	49.5	51.4

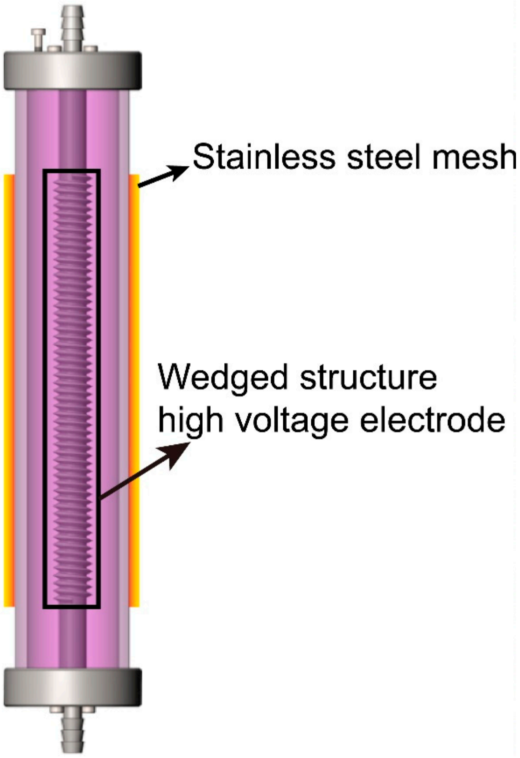

Figure S1. Enlarged illustration of the wedged high electrode and DBD reactor.

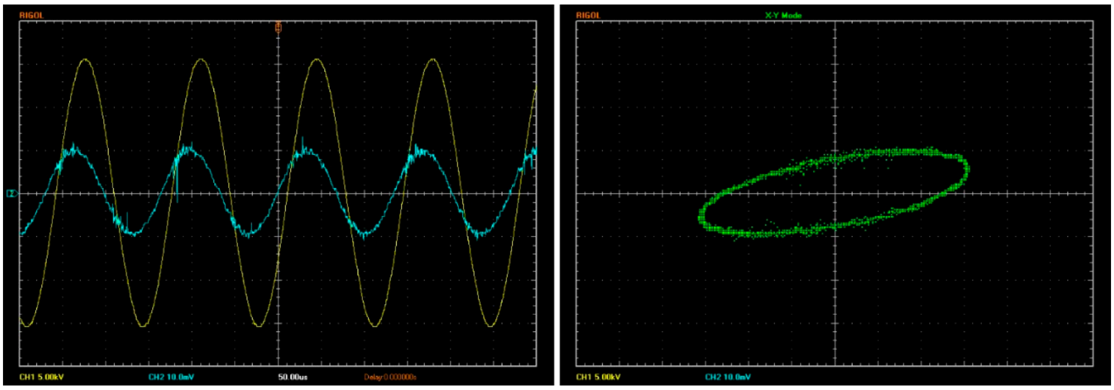

Figure S2. Waveforms of applied voltage and V-Q Lissajous diagrams of IPC process at 15 kV peak voltage.

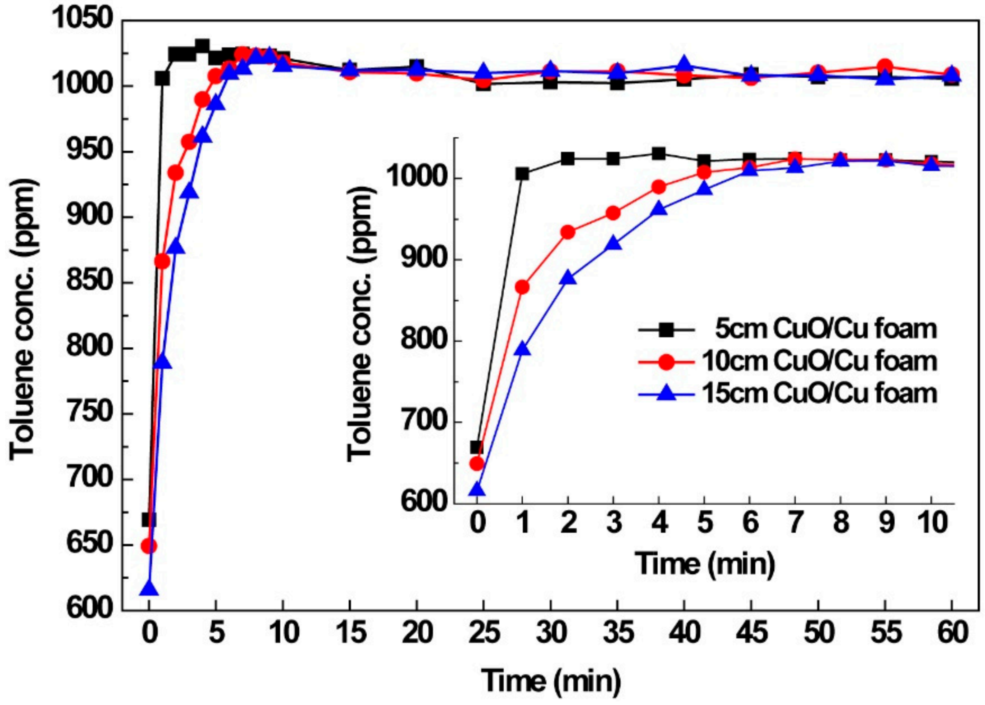

Figure S3. Toluene adsorption balance of different CuO foam loading in the IPC reactor.

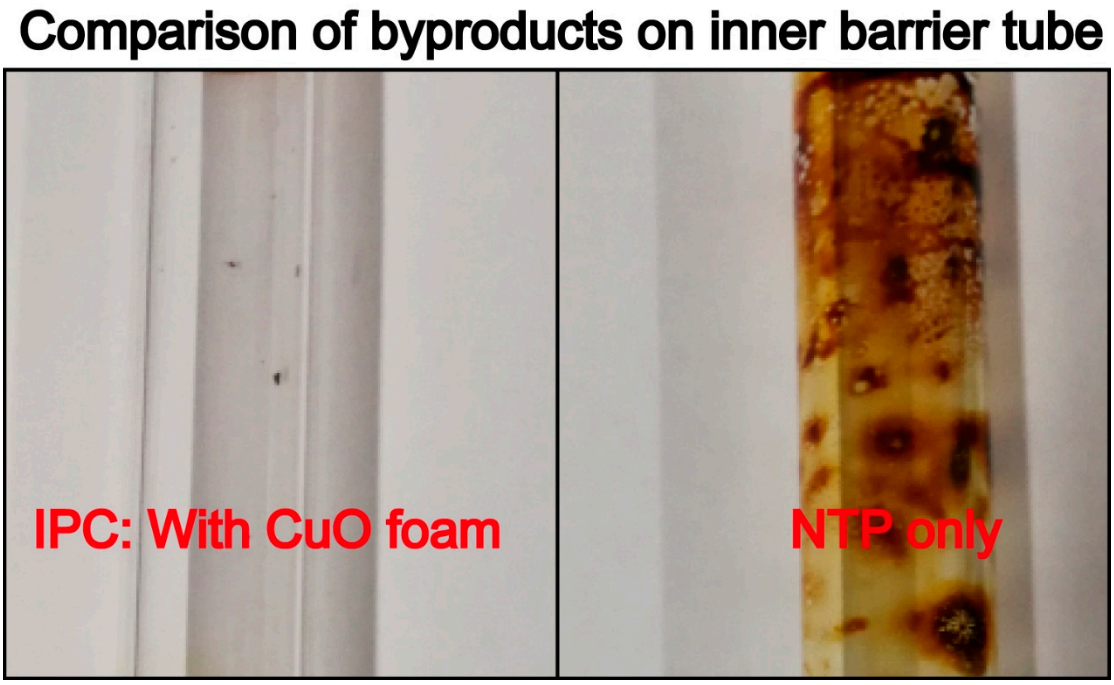

Figure 4. Toluene decomposition byproduct on inner barrier tube comparison of the NTP and IPC process.

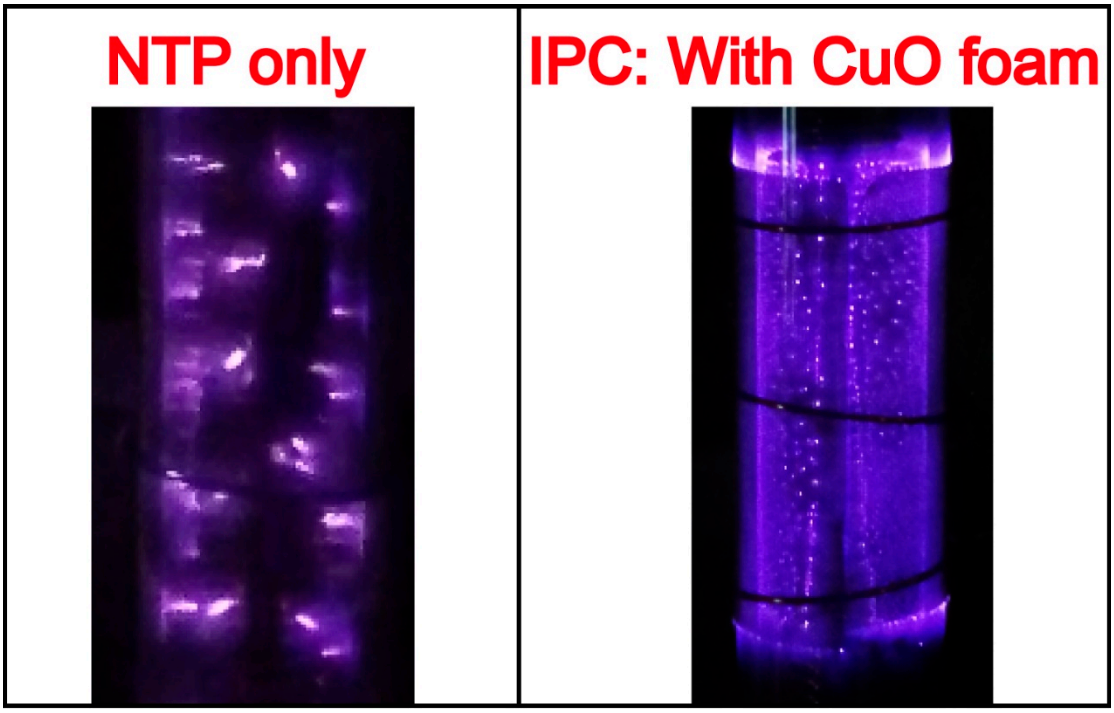

Figure 5. Discharge phenomenon of DBD process with (**right**) and in the absence of CuO foam (**left**) as a catalyst.
